# A (five-)level playing field for mental health conditions?: exploratory analysis of EQ-5D-5L-derived utility values

**DOI:** 10.1007/s11136-017-1768-1

**Published:** 2017-12-16

**Authors:** E. M. Camacho, G. Shields, K. Lovell, P. A. Coventry, A. P. Morrison, L. M. Davies

**Affiliations:** 1 Division of Population Health, Health Services Research, and Primary Care, School of Health Sciences, Centre for Health Economics, University of Manchester, Oxford Road, Manchester, M13 9PL UK; 2 Division of Nursing, Midwifery & Social Work, School of Health Sciences, University of Manchester, Manchester, UK; 3 Department of Health Sciences, University of York, York, UK; 4 Centre for Reviews and Dissemination, University of York, York, UK; 5 Division of Psychology & Mental Health, School of Health Sciences, University of Manchester, Manchester, UK

**Keywords:** Health-related quality of life, Mental health, Schizophrenia, Depression, Utility, EuroQol

## Abstract

**Purpose:**

Economic evaluations of mental health interventions often measure health benefit in terms of utility values derived from the EQ-5D. For the five-level version of the EQ-5D, there are two methods of estimating utility [crosswalk and stated preference (5L-SP)]. This paper explores potential impacts for researchers and decision-makers when comparing utility values derived from either method in the specific context of mental health.

**Methods:**

Baseline EQ-5D-5L data from three large randomised controlled trials of interventions for mental health conditions were analysed. Utility values were generated using each method. Mean utility values were compared using a series of *t* tests on pooled data and subgroups. Scenario analyses explored potential impacts on cost-effectiveness decisions.

**Results:**

EQ-5D data were available for 1399 participants. The mean utility value for each trial was approximately 0.08 higher when estimated using the 5L-SP approach compared to crosswalk (*p* < 0.0001). The difference was greatest among people reporting extreme anxiety/depression (mean utility 5L-SP 0.309, crosswalk 0.084; difference = 0.225; *p* < 0.0001). Identical improvements in health status were associated with higher costs to gain one QALY with the 5L-SP approach; this is more pronounced when improvements are across all domains compared to improvements on the anxiety/depression domain only.

**Conclusions:**

The two approaches produce significantly different utility values in people with mental health conditions. Resulting differences in cost per QALY estimates suggest that thresholds of cost-effectiveness may also need to be reviewed. Researchers and decision-makers should exercise caution when comparing or synthesising data from trials of mental health interventions using different utility estimation approaches.

## Introduction

In the UK, approximately one in four adults experience mental health problems in a given year [1]. The annual cost to society is estimated to be £70–100 bn (20% from health and social care costs, 30% from lost productivity, and 50% from human suffering) [2]. Mental illness is the largest category of NHS disease expenditure and accounts for 28% of the total burden of disease in the UK [2].

Mental health disorders have a negative impact on health-related quality of life (HRQoL) which varies according to the specific diagnosis [3, 4]. There are many ways in which mental health problems can impact HRQoL, for instance feelings of hopelessness or anxiety, low self-esteem, a lack of confidence, loneliness, and feeling a lack of control [5].

The EuroQol 5 dimension questionnaire (EQ-5D) is a short, self-report measure used to assess health status over five domains: mobility, self-care, ability to do usual activities, pain/discomfort, and anxiety/depression [6, 7]. The EQ-5D-3L asks respondents to rate their health at one of three levels (see Box 1) for each of the health domains. This produces 243 possible profiles of health. In the 1990s, a series of time-trade-off (TTO) exercises were conducted to generate a country-specific health state index value for each of the health profiles (e.g. [8] in the UK). These values correspond to how favourable or unfavourable each health state is viewed by the general population of a particular country. They are used in economic evaluations of healthcare interventions to quantify health utility and combined with life expectancy to calculate quality-adjusted life-years (QALYs) for cost–utility analyses, as recommended by the National Institute for Health and Care Excellence (NICE) in England [9].

There has been some debate over whether utility values derived from the EQ-5D-3L are sensitive to important clinical health improvements for people with mental health conditions as only one health domain directly measures mental health [10, 11]. A 5-level version of the EQ-5D has now been published which aims to improve on the 3-level design, making it more sensitive to smaller changes in health. In the 5L version, there are five possible responses along the same best-to-worst scale as the 3-level version (see Box 1), producing 3125 possible profiles of health [7].

**Box 1:** Responses on the EQ-5D three- and five-level versions

Three-level	Five-level
No problems	No problems
	Slight problems
Some problems	Moderate problems
	Severe problems
Extreme problems	Extreme problems

When the EQ-5D-5L was first released, a probability-based, non-parametric, mapping exercise was conducted to produce a set of utility values from the 3L value set (i.e. restricted to the same range) [12]. This is referred to as the crosswalk approach. In 2017, the results were published from an exercise combining two stated preference (SP) methods, TTO and discrete choice experiment (DCE) [13], to derive an EQ-5D-5L utility value set for England [14]. This will subsequently be referred to as the 5L-SP approach.

It is known that comparable improvements in health status are measured as larger gains in health utility with the 3L than the 5L version [15]. This is the combined effect of the different number of levels, valuation protocol, and range/distribution of possible utility values. The valuation protocol and range/distribution of utility values are potential mechanisms for how differences may arise between EQ-5D-5L utility values estimated using the 5L-SP and crosswalk approaches [16].

These differences may have specific implications for people with mental health conditions. For the crosswalk utility values, anxiety/depression is the third most important domain (size of the level 5 coefficient), whereas for the 5L-SP values anxiety/depression is the second most important domain [14]. Furthermore, the TTO exercise for England found that people did not differentiate between severe (level 4) and extreme (level 5) anxiety/depression as had been expected [13]. This was somewhat corrected for by the hybrid TTO and DCE approach. However, an improvement in anxiety/depression from ‘extreme’ to ‘severe’, which may represent an important improvement for an individual experiencing these health states, is still associated with a smaller QALY gain than other one-level improvements on this domain.

There may be important implications of these differences when comparing findings from studies of mental health interventions which have used different versions and approaches for estimating utility. An important strength of the crosswalk approach for the 5L version is that because utility values directly map onto those generated from the 3L version, in theory results can be compared between studies using the different versions. Whereas the differences in the methods used mean, it is not appropriate to use 5L-SP and 3L-TTO utility values interchangeably [15].

## Methods

The aim of this analysis is to calculate utility values derived from the EQ-5D-5L using each of the two approaches to explore potential implications for researchers and decision-makers. Mental health may be considered a special case in relation to the EQ-5D and so this aim was addressed in the context of three large randomised controlled trials (RCTs) of interventions for mental health conditions.

Our key research questions are as follows:

1. How do utility values differ between the two methods (5L-SP and crosswalk) used to estimate utility from the EQ-5D-5L?
2. How do EQ-5D-5L responses and utility values differ across three study samples with different mental health conditions?
3. What impact does the method of utility estimation have on estimates of cost-effectiveness?

The studies were selected from existing datasets held by the authors for trials which collected baseline EQ-5D-5L and were available at the time of the analysis. Protocols for the respective studies describe the methods completely [17–19].

The key details are as follows:

- COINCIDE—COlaborative INterventions for CIrculation and DEpression [17]
  - Population—Adults with diabetes and/or coronary heart disease with comorbid depression
  - Sample—*n* = 387, 38% female, mean age 59 years.
- EQUIP—Enhancing the Quality of User Involved care Planning in mental health services [18]
  - Population—A mixed population of adults with a severe mental illness (including diagnoses of schizophrenia, bipolar disorder, and depression) accessing secondary care mental health services.
  - Sample—*n* = 602, 60% female, mean age 55 years.
- FOCUS—Focusing On Clozapine Unresponsive Symptoms [19]
  - Population—People aged at least 16 years with confirmed treatment-resistant schizophrenia that is poorly responsive to an adequate trial of clozapine monotherapy
  - Sample—*n* = 487, 28% female, mean age 43 years.

This analysis was restricted to baseline EQ-5D-5L data as follow-up data were not available for all three studies at the time of the analysis. Utility values were calculated from the EQ-5D-5L using the crosswalk approach [12] and the 5L-SP approach [14].

Unless stated otherwise, the analyses were conducted on data from all three studies simultaneously (pooled data). This means that the proportionate differences between utility values calculated using the two approaches were explored in the largest dataset possible, irrespective of differences in the characteristics of the different trial samples. The responses for the EQ-5D-5L were summarised graphically using the eq5dds command in STATA [20].

Descriptive statistics [mean, standard deviation (SD), range, 95% confidence interval (CI)] were used to summarise the utility values estimated by each approach. The rationale for adjusting for confounders in statistical analyses is that confounders have an effect on both the independent (input) and dependent (outcome) variables in a relationship. For the analyses reported here, there are no input variables, and only an outcome variable (utility) was calculated in two different ways. As such, the statistical approach involved direct comparison of unadjusted mean values. *T* tests were used to evaluate whether differences in the mean utility values produced by the alternative approaches were significant.

### Subgroup analysis

To further explore the ‘mental health’ domain of the EQ-5D-5L, subgroups were defined according to the level of anxiety/depression. Mean utility values were compared across the subgroups.

### Scenario analysis

To explore the possible implications of the different utility estimation methods on cost-effectiveness estimates, pseudo follow-up EQ-5D-5L profiles were generated for all participants from their baseline values. This was done for two different scenarios of health status improvement (scenario 1: 1-level improvement on the anxiety/depression domain; scenario 2: 1-level improvement on all domains). For example, a participant with a baseline profile of 12,345 would have a ‘follow-up’ profile of 12,344 in scenario 1 and 11,234 in scenario 2. As with real-world follow-up data, under both scenarios it was not possible to ‘improve’ beyond level 1 on any domain. This ensured that the pseudo follow-up profiles related directly to the utility value sets for both the crosswalk and SP approaches. ‘Follow-up’ utility values were then calculated, in the same way as baseline values, using each estimation method. QALY gains were then estimated for each individual by subtracting their baseline utility value from their follow-up value, assuming a 1-year time horizon. The mean QALY gain across the whole sample (pooled dataset) was used to calculate incremental cost-effectiveness ratios (ICERs) for each scenario over a range of costs (£500, £1000, £5000, and £10,000).

All analyses were conducted on a complete case basis using the STATA software program (StataCorp. 2013. Stata Statistical Software: Release 13. College Station, TX: StataCorp LP).

## Results

The characteristics of the pooled sample are reported in Table 1. The pooled sample had a mean age of 48 years, just over half were male, and the majority were of white ethnicity. The most common level of education within the sample was compulsory secondary education (approximately age 16). Approximately one-fifth of the sample were in paid employment (full or part time). The total sample size was 1476, 1399 (95%) of whom had completed the EQ-5D-5L.

**Table 1:** Baseline characteristics of the pooled sample from the three trials

*n* = 1476	Mean (SD) or *n*/*N* (%)
Age (years)	48 (13.2); *n* = 1462
Sex (female)	640/1461 (44%)
Ethnicity (white)	1244/1472 (85%)
Education*	
Secondary school	453/1023 (44%)
Further education	274/1023 (27%)
Higher education	296/1023 (29%)
Employment status (in paid employment)**	178/963 (18%)

*Data available for FOCUS and EQUIP trials only (*n* = 1089)

**Data available for COINCIDE and EQUIP trials only (*n* = 989)

**Fig. 1:**
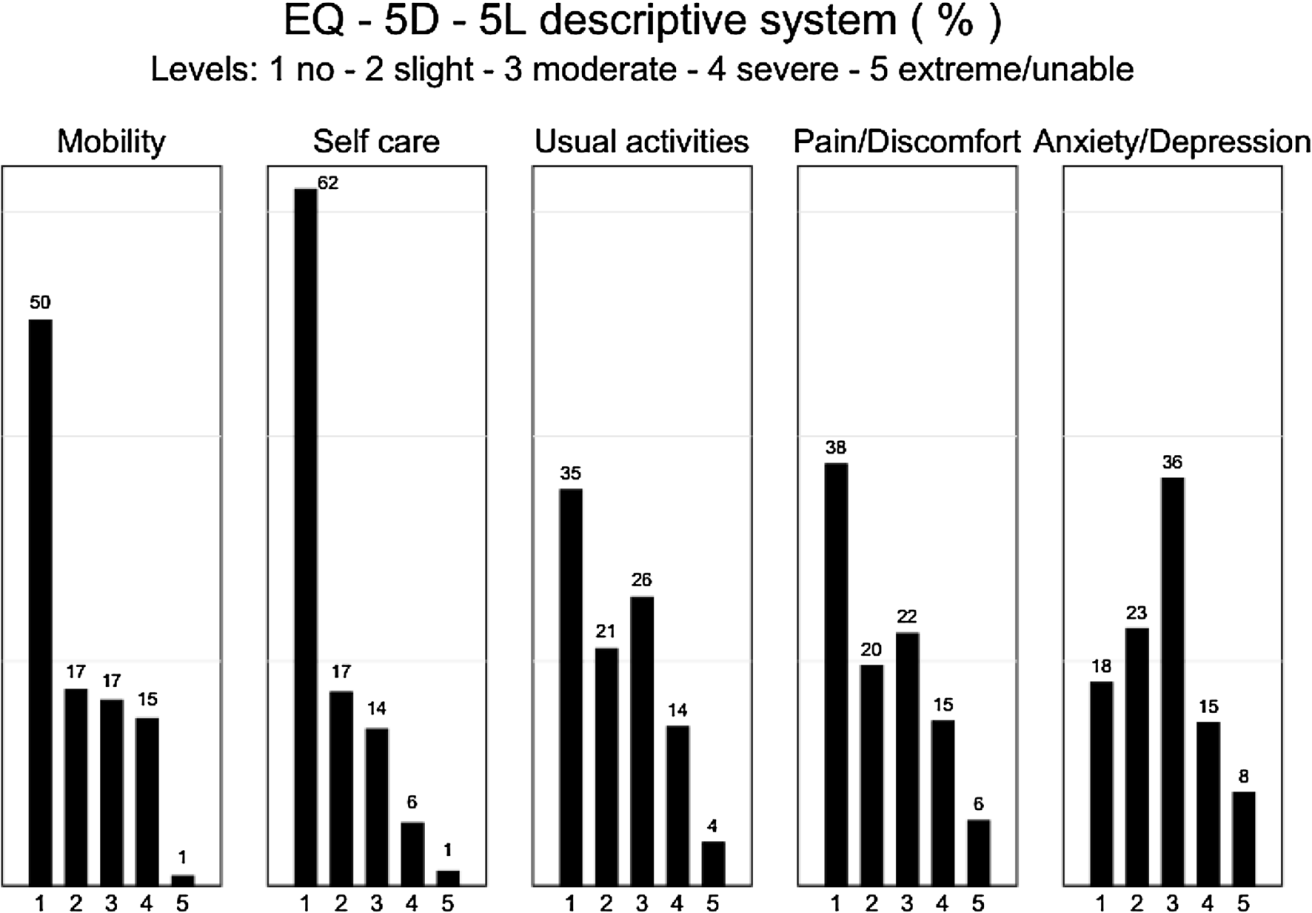
Distribution of responses on EQ-5D-5L questionnaire (pooled data)

Figure 1 shows the distribution of EQ-5D-5L responses for each domain (pooled data). Whilst the most common response on the first four domains is ‘no problems’, the anxiety and depression domain peaks at ‘moderate problems’ and shows less variation across the five levels.

**Fig. 2:**
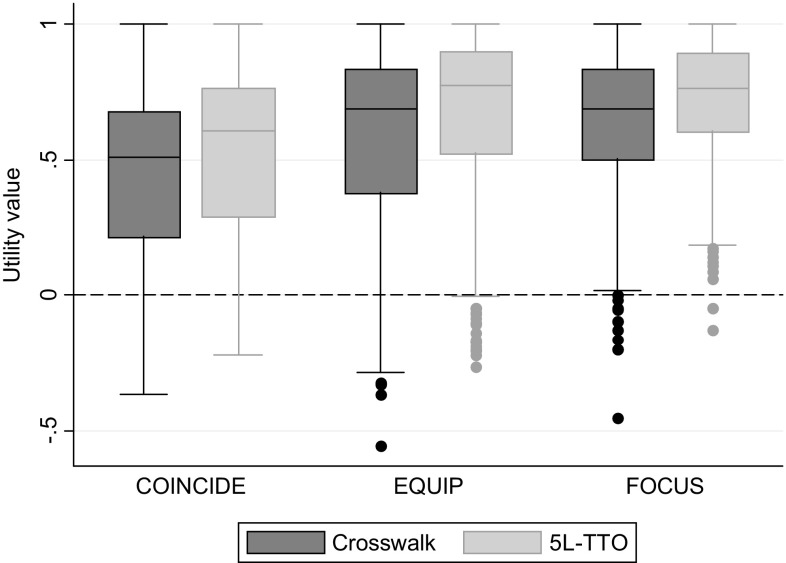
Utility values for the original study samples, by utility estimation approach

Table 2 and Fig. 2 show the calculated EQ-5D-5L utility values using both methods. For both the pooled data and each trial separately, the difference in the mean utility value derived using the 5L-SP approach was approximately 0.08 higher than the crosswalk value, a statistically significant difference (*p* < 0.05).

**Table 2:** Summary statistics for utility values estimated using the alternative approaches

EQ-5D-5L utility	Mean (SD)	Min	Max	95% CI
Pooled data (*n* = 1399)				
5L-SP	0.644 (0.28)	− 0.263	1	0.629–0.659
Crosswalk	0.565 (0.31)	− 0.555	1	0.549–0.581
Mean difference (*p* value)	0.079 (*p* < 0.0001)			
COINCIDE (*n* = 366)				
5L-SP	0.521 (0.29)	− 0.218	1	0.491–0.551
Crosswalk	0.449 (0.29)	− 0.367	1	0.419–0.479
Mean difference (*p* value)	0.072 (*p* < 0.0001)			
EQUIP (*n* = 580)				
5L-SP	0.667 (0.30)	− 0.263	1	0.643–0.692
Crosswalk	0.587 (0.34)	− 0.555	1	0.559–0.614
Mean difference (*p* value)	0.081 (*p* < 0.0001)			
FOCUS (*n* = 453)				
5L-SP	0.714 (0.22)	− 0.127	1	0.694–0.734
Crosswalk	0.631 (0.26)	− 0.453	1	0.608–0.655
Mean difference (*p* value)	0.082 (*p* < 0.0001)			

Participants in the COINCIDE trial (depression alongside a long-term physical condition) had the lowest mean utility values; this group also had the oldest average age. The highest mean utility value came from the FOCUS trial (schizophrenia). Both the EQUIP and FOCUS trials included participants with serious mental illness (schizophrenia is included in this broader umbrella term) which means that they could plausibly be used to inform parameters in the same economic model. When the same utility estimation method is used, the mean baseline utility is higher for the FOCUS trial than for EQUIP by between 0.044 (crosswalk) and 0.0447 (5L-SP). However, if baseline utility was reported using the 5L-SP values for FOCUS and the crosswalk values for EQUIP, the FOCUS utility values would be higher by 0.127 (i.e. 0.714 − 0.587 = 0.127), and if the other utility value was used in each case, the EQUIP utility values would be higher by 0.036 (i.e. 0.667 − 0.631 = 0.036).

Table 3 shows the mean utility values derived using each approach for subgroups of the pooled dataset defined by participants’ response on the anxiety/depression domain of the EQ-5D-5L. By far, the greatest difference in utility values calculated using the different methods is seen in those who reported being extremely anxious or depressed, 8% of the pooled dataset.

**Table 3:** Mean utility values (pooled data) by response on anxiety/depression domain of EQ-5D-5L and utility values from published tariffs for different health profiles

*n*/*N* (%)	5L-SP	Crosswalk	Difference (5L-SP–crosswalk)	*p* value for difference
Not anxious or depressed [253/1399 (18%)]	0.885	0.829	0.057	< 0.0001
Slightly anxious or depressed [320/1399 (23%)]	0.740	0.669	0.070	< 0.0001
Moderately anxious or depressed [507/1399 (36%)]	0.642	0.574	0.069	< 0.0001
Severely anxious or depressed [203/1399 (15%)]	0.388	0.325	0.063	< 0.0001
Extremely anxious or depressed [116/1399 (8%)]	0.309	0.084	0.225	< 0.0001

Table 4 reports ICERs for a hypothetical intervention associated with either a 1-level improvement from baseline in anxiety/depression or a 1-level improvement from baseline on all EQ-5D-5L domains. In both of these instances, the 5L-SP approach estimates a higher cost to gain one QALY than the crosswalk approach. This reflects how an identical change in health status is associated with a smaller improvement in utility value according to the 5L-SP approach rather than the crosswalk approach.

**Table 4:** ICERs calculated at different levels of net cost for two scenarios of health improvement applied to the pooled dataset; values are cost to gain one QALY (assuming utility values are accrued over 1 year)

Health status improvement	Cost	ICER (£/QALY gained)		Difference
		5L-SP	Crosswalk	
1-level improvement in anxiety/ depression^a^	£500	£9259	£8197	£1062
	£1000	£18,519	£16,393	£2126
	£5000	£92,593	£81,967	£10,626
	£10,000	£185,185	£163,934	£21,251
1-level improvement on each EQ-5D domain^b^	£500	£2747	£2427	£320
	£1000	£5495	£4854	£641
	£5000	£27,473	£24,272	£3201
	£10,000	£54,945	£48,544	£6401

^a^Mean QALY gain in sample (pooled data): 5L-SP 0.054; crosswalk 0.061; difference 0.007

^b^Mean QALY gain in sample (pooled data): 5L-SP 0.182; crosswalk 0.206; difference 0.024

If a decision-maker used a cost-effectiveness threshold of £17,000 per QALY gained, then the intervention improving anxiety/depression by one level, at a net cost of £1000, would only be considered cost-effective according to the crosswalk approach (Table 4).

A 1-year time horizon has been assumed for these analyses; however, there would be a multiplicative effect over time (i.e. a utility value of 0.08 would equate to 0.08 QALYs over 1 year, 0.16 QALYs over 2 years, and 0.80 QALYs over 10 years).

## Discussion

This analysis provides EQ-5D-5L scores and utility values for three large mental health trials. The utility values estimated according to the two approaches were significantly different from each other. The 5L-SP approach estimated utility values approximately 0.08 higher than the crosswalk approach for the same health profiles.

Another comparison of utility values estimated using the two approaches, albeit for a range of different health conditions, reported a similar mean difference in utility of around 0.09 [16]. Our findings also support previous analyses which demonstrated how comparable improvements in health are measured as larger using the 3L (i.e. the same value set at the crosswalk approach) and 5L versions of the EQ-5D [15].

In the analysis reported here, the difference between 5L-SP and crosswalk utility values was around three times the size among participants reporting level 5 anxiety/depression than for those reporting any other level. This corresponds to the finding reported by the authors who derived the TTO utility tariffs for the EQ-5D-5L that people did not differentiate between level 4 and level 5 anxiety/depression as had been expected during the TTO exercise [13].

Comparing the different trials included in this analysis showed that as long as the same utility estimation method is used for both samples, similar differences between the mean utility values for FOCUS and EQUIP are found (FOCUS had higher utility with both methods—0.044 (crosswalk) and 0.047 (5L-SP)). However, comparing the values generated by the different utility estimation methods demonstrated how conflicting results could occur. For example, EQUIP utility values were higher than the FOCUS values when the 5L-SP approach was used for EQUIP and the crosswalk approach for FOCUS.

Scenario analyses confirmed that an intervention may be less likely to be classified as cost-effective (same health improvement associated with a lower QALY gain and thus a higher ICER) using the 5L-SP method compared with the crosswalk method.

### Strengths and weaknesses

A key strength of this analysis is the high quality of the data sources. The data come from three large, robust trials of different mental health conditions. The level of missing data is minimal with 95% of participants recruited to the trials completing the EQ-5D at baseline.

One limitation of this analysis is that the TTO value set for the EQ-5D-5L was from a sample of the population living in England and thus the research is specific to England and findings may not be generalisable to other countries. Furthermore, the findings may not be relevant to mental health conditions that were not included here.

Another limitation that can be addressed in future work is that this analysis included only baseline data from the studies and so it was not possible to explore differences in ICERs. It will be interesting to examine this when follow-up data are available.

### Potential implications

There are two key areas for which these findings may have potentially important implications: decision-making and evidence synthesis. In terms of decision-making, the method of utility estimation could determine whether an ICER falls above or below a particular cost-effectiveness threshold. Assuming that the same threshold for cost-effectiveness is applied, it is possible that interventions may be considered cost-effective using the crosswalk method but may not using the 5L-SP method. Results from this analysis suggest that the impact of this is likely to be greatest for samples with a large proportion of people reporting extreme anxiety/depression.

Evidence synthesis involves bringing together estimates of costs, benefits (QALYs), and/or ICERs from a range of sources (e.g. systematic reviews, meta-analyses). EQ-5D-5L data calculated using the crosswalk method are at present most likely to be identified by systematic reviews, as these have been available for longer. Evidence synthesis is also often part of the economic decision modelling process. As shown here using the cautionary example of the EQUIP and FOCUS studies, which, because of the overlapping mental health conditions they include, could potentially be included in the same decision model, it is important that researchers are aware that combining utility values estimated using different approaches is not necessarily straightforward. Economic models are also used to extrapolate findings from RCTs over longer periods, and there may also be specific implications of utility estimation method in these models as differences are multiplied over time.

A recommendation for future economic evaluations using the EQ-5D-5L would be to ensure that results according to both methods of utility estimation are reported, or at least that the method of utility estimation is clearly reported in publications of the results.

This analysis will be of particular interest to decision-making bodies, such as the National Institute for Health and Care Excellence (NICE). The Decision Support Unit (DSU) for NICE reported that there may be implications of the move from the 3-level to the 5-level version of the EQ-5D for the thresholds used to evaluate cost-effectiveness [15]. This introduces another issue to the ongoing debate over the threshold at which an intervention should be considered to be cost-effective in the UK, currently argued to be anywhere between £12,000 and £30,000 per QALY gained [21–24]. To ensure that an intervention has the same likelihood of being classified as cost-effective, regardless of which approach is used to generate utility values from the EQ-5D-5L, it may be appropriate to define a different threshold for each approach. This may be less of an issue when comparing the two methods of utility estimation for the 5-level version because the difference in the distribution of values for 5L-SP versus crosswalk is smaller than that for 5L-SP versus 3L-TTO [16].

In conclusion, the differences in EQ-5D-5L-derived utility values estimated using the crosswalk and 5L-SP approaches appear to be broadly similar among samples with mental health conditions and other health conditions. There are implications of utility estimation approach for decision-making and comparing and combining data from different studies. The implications are likely to be greatest for people reporting extreme anxiety/depression and evaluations over long time horizons. Updated guidance from NICE and other bodies for how this should be reported and implications handled in terms of health technology assessment is needed.
